# Supplementary material for: Analysis of University Students’ Mental Health from the Perspective of Occupational Harmony
Source: PLoS One. 2025 Apr 1;20(4):e0309490. doi: 10.1371/journal.pone.0309490 (PMC11960997; doi:10.1371/journal.pone.0309490)
Supplement: S2 Table — OHQ: The Occupational Harmony Questionnaire, DASS-21: The Depression Anxiety Stress Scale. Participant experience content areas. a: Characteristics of occupational engagement; b: Overall statement on mental health. Participants A to K participated in individual interviews. Participants L to O participated in a focus group discussion. (DOCX) [file pone.0309490.s002.docx]

**S2 Table. Profiles of 15 Participants in the Qualitative Study**

| No. | Gender | Grade | OHQ subjective scale score | DASS-21 results | Prior mental health issues | Occupational engagement and mental health status  (from interviews and a focus group discussion) |
| --- | --- | --- | --- | --- | --- | --- |
| A | Female | Senior | 78.4 | Normal | No | 1. Lacked motivation and planning for study, had challenges in the internship, stayed up late and lacked exercise, limited social activity but got along well with family members 2. Felt isolated and bored, worried about taking exams, hard to concentrate |
| B | Male | Sophomore | 72.8 | Normal | No | 1. Had nothing to do at home and had conflicts with family members, hard to complete assignments at school 2. Felt bored and annoyed about family members’ demands, anxious about academic pressure |
| C | Female | Sophomore | 71.2 | Normal | No | 1. Lack of planning and stayed up late to work on assignments, excessive entertainment at home, lacked exercise 2. Enjoyed leisure time but felt stressed and anxious about studying, worried about physical health |
| D | Female | Sophomore | 77.6 | Normal | No | 1. Very busy completing coursework and stayed up late, addicted to smartphones during home isolation, lack of exercise, had conflicts with family members 2. Felt lonely and stressed about study |
| E | Female | Master’s | 61.6 | Mild anxiety | No | 1. Lack of planning and procrastinated in research, stayed up late, had conflicts with family members 2. Felt lonely and anxious about meeting graduation requirements |
| F | Female | Junior | 73.6 | Normal | No | 1. Canceled an overseas exchange plan, got along well with family members at home, stayed up late and busy with research projects at school, lacked exercise 2. Felt a bit depressed but enjoyed time with family members during home isolation, felt anxious about academic competition |
| G | Female | Senior | 53.6 | Mild anxiety | No | 1. Had nothing to do at home, spent time on screen over 10 h/d, had conflicts with parents and ran away from home 2. Felt meaningless and anxious, worried about the graduate entrance exam |
| H | Female | Senior | 77.6 | Normal | No | 1. Bauge-watching and staying up late during home isolation, had conflicts with family members, lack of planning and lost the qualification for postgraduate recommendations 2. Felt lonely at home, and very regretful about her academic failure |
| I | Female | Senior | 65.6 | Severe anxiety, mild depression, mild stress | No | 1. Canceled plans to study abroad, ate too much fast food, had difficulty connecting with others 2. Felt devastated, isolated, and worried about her health and family members |
| J | Male | Doctorate | 51.2 | Normal | Yes | 1. Slept too much (over 10-11h/d), lack of planning and was unable to work on the research project, limited participation in collective activities 2. Felt afraid and worried about graduation |
| K | Female | Sophomore | 47.2 | Severe anxiety, severe depression, mild stress | No | 1. Bauge-reading and staying up late all night during home isolation, lack of motivation to study, limited communication with others 2. Felt depressed, had suicidal thoughts |
| L | Male | Junior | 70.4 | Normal | No | 1. Lacked clear goals and plans for study, canceled travel plans, irregular sleep pattern 2. Felt a bit anxious about future |
| M | Female | Master’s | 79.2 | Normal | No | 1. Conctrented on graduation thesis, participated in some leisure activities (e.g., watching movies, jigsaw puzzles) 2. Felt balanced and peaceful |
| N | Female | Master’s | 62.4 | Severe anxiety, severe depression, moderate stress | Yes | 1. Slept and rested over 12 h/d, read many books at home, lack of planning and had difficulty working on thesis 2. Enjoyed life at home but felt anxious and stressed after returning to school |
| O | Female | Master’s | 75.2 | Normal | No | 1. Canceled plans to study abroad, had difficulty finding a job, concentrated on study and work, regular exercise, got along well with family members 2. Felt miserable and unclear about future plans initially but got back to normal soon |

OHQ: The Occupational Harmony Questionnaire, DASS-21: The Depression Anxiety Stress Scale. Participant experience content areas. a: Characteristics of occupational engagement; b: Overall statement on mental health. Participants A to K participated in individual interviews. Participants L to O participated in a focus group discussion.
