# Supplementary material for: Analysis of University Students’ Mental Health from the Perspective of Occupational Harmony
Source: PLoS One. 2025 Apr 1;20(4):e0309490. doi: 10.1371/journal.pone.0309490 (PMC11960997; doi:10.1371/journal.pone.0309490)
Supplement: Attachment — Submitted filename: PONE-D-24-34209.pdf [file pone.0309490.s003.pdf]

PLOS ONE

Analysis of university students' mental health from the perspective of occupational harmony

--Manuscript Draft--

**Manuscript Number:**	PONE-D-24-34209
**Article Type:**	Research Article
**Full Title:**	Analysis of university students' mental health from the perspective of occupational harmony
**Short Title:**	Analysis of university students' mental health from the perspective of occupational harmony
**Corresponding Author:**	Yijun Liu<br>Peking University First Hospital<br>Beijing, Beijing CHINA
**Keywords:**	occupational engagement; occupational harmony; mental health; university students; COVID-19 Pandemic
**Abstract:**	Background: Mental health problems are common among university students. Based on the Model of Occupational Harmony, we investigated the relationship between occupational engagement and mental health among Chinese university students.<br>Methods: A mixed methods approach was adopted. A total of 734 Chinese university students responded to online questionnaires: a socio-demographic questionnaire, the Depression Anxiety Stress Scale, the WHOQOL-BREF, and a self-designed Occupational Harmony Questionnaire. Individual interviews with 11 university students and a focus group of four students provided qualitative data.<br>Results: Anxiety, depression, and stress were reported by 11.9%, 9.4%, and 2.9% of the participants, respectively. The level of occupational harmony was negatively correlated with depression, anxiety, and stress ($p < .001$). The DASS-21 score was significantly different among students who spent different amounts of time on the following activities: exercise, sleep, rest, reflective and consulting activities, study, work, and leisure and entertainment ($P < .05$). In the context of the COVID-19 pandemic, the key occupational factors contributing to university students' mental health problems included excessive mental engagement and insufficient physical engagement, excessive active engagement and insufficient quiet engagement, insufficient social engagement, excessive or insufficient Gong (productive) engagement, and insufficient De (virtuous) engagement, Zhi (rational) engagement, and Kang (health maintenance) engagement, lack of coherence within the family, school, social systems, as well as inadequate schedule planning and time management.<br>Conclusions: Occupational disharmony is related to university students' mental health problems. Occupational therapy can promote university students' mental well-being by enhancing occupational harmony.
**Order of Authors:**	Yijun Liu, Doctor
	Ruiqi She, Bachelor
	Jing Xin, Master
**Additional Information:**	
**Question**	**Response**
**Financial Disclosure**<br><br>Enter a financial disclosure statement that describes the sources of funding for the work included in this submission. Review the submission guidelines for detailed requirements. View published research	Yes

Powered by Editorial Manager® and ProduXion Manager® from Aries Systems Corporation

articles from *PLOS ONE* for specific examples.<br><br>This statement is required for submission and **will appear in the published article** if the submission is accepted. Please make sure it is accurate.<br><br>**Funded studies**<br>Enter a statement with the following details:<br>• Initials of the authors who received each award<br>• Grant numbers awarded to each author<br>• The full name of each funder<br>• URL of each funder website<br>• Did the sponsors or funders play any role in the study design, data collection and analysis, decision to publish, or preparation of the manuscript?<br><br>Did you receive funding for this work?	
Please add funding details.<br>as follow-up to "**Financial Disclosure**<br><br>Enter a financial disclosure statement that describes the sources of funding for the work included in this submission. Review the submission guidelines for detailed requirements. View published research articles from *PLOS ONE* for specific examples.<br><br>This statement is required for submission and **will appear in the published article** if the submission is accepted. Please make sure it is accurate.	National High Level Hospital Clinical Research Funding (Youth Clinical Research Project of Peking University First Hospital) 2024YC10

Powered by Editorial Manager® and ProduXion Manager® from Aries Systems Corporation

**Funded studies**<br>Enter a statement with the following details:<br>• Initials of the authors who received each award<br>• Grant numbers awarded to each author<br>• The full name of each funder<br>• URL of each funder website<br>• Did the sponsors or funders play any role in the study design, data collection and analysis, decision to publish, or preparation of the manuscript?<br><br>Did you receive funding for this work?"	
Please select the country of your main research funder (please select carefully as in some cases this is used in fee calculation).<br>as follow-up to "**Financial Disclosure**<br><br>Enter a financial disclosure statement that describes the sources of funding for the work included in this submission. Review the submission guidelines for detailed requirements. View published research articles from *PLOS ONE* for specific examples.<br><br>This statement is required for submission and **will appear in the published article** if the submission is accepted. Please make sure it is accurate.<br><br>**Funded studies**<br>Enter a statement with the following details:<br>• Initials of the authors who received each award<br>• Grant numbers awarded to each author<br>• The full name of each funder<br>• URL of each funder website<br>• Did the sponsors or funders play any role in the study design, data collection and analysis, decision to publish, or preparation of the manuscript?<br><br>Did you receive funding for this work?"	CHINA - CN
**Competing Interests**	NO authors have competing interests

Use the instructions below to enter a competing interest statement for this submission. On behalf of all authors, disclose any competing interests that could be perceived to bias this work—acknowledging all financial support and any other relevant financial or non-financial competing interests.<br><br>This statement is required for submission and **will appear in the published article** if the submission is accepted. Please make sure it is accurate and that any funding sources listed in your Funding Information later in the submission form are also declared in your Financial Disclosure statement.<br><br>View published research articles from *PLOS ONE* for specific examples.<br><br>**NO authors have competing interests**<br><br>Enter: *The authors have declared that no competing interests exist.*<br><br>**Authors with competing interests**<br><br>Enter competing interest details beginning with this statement:<br><br>*I have read the journal's policy and the authors of this manuscript have the following competing interests: [insert competing interests here]*<br><br>* typeset	
**Ethics Statement**<br><br>Enter an ethics statement for this submission. This statement is required if the study involved:<br><br>• Human participants<br>• Human specimens or tissue<br>• Vertebrate animals or cephalopods<br>• Vertebrate embryos or tissues	Ethical approval was obtained from the ethics committee of Peking University First Hospital (Approval number: 2020Study104) and all participants agreed to the written informed consent prior to participation.

Powered by Editorial Manager® and ProduXion Manager® from Aries Systems Corporation

• Field research<br><br>Write "N/A" if the submission does not require an ethics statement.<br><br>General guidance is provided below. Consult the submission guidelines for detailed instructions. **Make sure that all information entered here is included in the Methods section of the manuscript.**	

**Format for specific study types**<br><br>**Human Subject Research (involving human participants and/or tissue)**<br>• Give the name of the institutional review board or ethics committee that approved the study<br>• Include the approval number and/or a statement indicating approval of this research<br>• Indicate the form of consent obtained (written/oral) or the reason that consent was not obtained (e.g. the data were analyzed anonymously)<br><br>**Animal Research (involving vertebrate animals, embryos or tissues)**<br>• Provide the name of the Institutional Animal Care and Use Committee (IACUC) or other relevant ethics board that reviewed the study protocol, and indicate whether they approved this research or granted a formal waiver of ethical approval<br>• Include an approval number if one was obtained<br>• If the study involved *non-human primates*, add *additional details* about animal welfare and steps taken to ameliorate suffering<br>• If anesthesia, euthanasia, or any kind of animal sacrifice is part of the study, include briefly which substances and/or methods were applied<br><br>**Field Research**<br><br>Include the following details if this study involves the collection of plant, animal, or other materials from a natural setting:<br>• Field permit number<br>• Name of the institution or relevant body that granted permission	
**Data Availability**<br><br>Authors are required to make all data underlying the findings described fully available, without restriction, and from the time of publication. PLOS allows rare exceptions to address legal and ethical concerns. See the PLOS Data Policy and FAQ for detailed information.	Yes - all data are fully available without restriction

A Data Availability Statement describing where the data can be found is required at submission. Your answers to this question constitute the Data Availability Statement and **will be published in the article**, if accepted.<br><br>**Important:** Stating 'data available on request from the author' is not sufficient. If your data are only available upon request, select 'No' for the first question and explain your exceptional situation in the text box.<br><br>Do the authors confirm that all data underlying the findings described in their manuscript are fully available without restriction?	
**Describe where the data may be found in full sentences. If you are copying our sample text, replace any instances of XXX with the appropriate details.**<br><br>• If the data are **held or will be held in a public repository**, include URLs, accession numbers or DOIs. If this information will only be available after acceptance, indicate this by ticking the box below. For example: *All XXX files are available from the XXX database (accession number(s) XXX, XXX.*).<br>• If the data are all contained **within the manuscript and/or Supporting Information files**, enter the following: *All relevant data are within the manuscript and its Supporting Information files.*<br>• If neither of these applies but you are able to provide **details of access elsewhere**, with or without limitations, please do so. For example:<br><br>*Data cannot be shared publicly because of [XXX]. Data are available from the XXX Institutional Data Access / Ethics Committee (contact via XXX) for researchers who meet the criteria for access to confidential data.*<br><br>*The data underlying the results presented in the study are available from (include the name of the third party*	All relevant data are within the manuscript and its Supporting Information files.

and contact information or URL).<br>• This text is appropriate if the data are owned by a third party and authors do not have permission to share the data.<br><br>* typeset	
Additional data availability information:	

Powered by Editorial Manager® and ProduXion Manager® from Aries Systems Corporation

Manuscript
Click here to access/download;Manuscript;Manuscript.docx

Analysis of University Students' Mental Health from the Perspective of Occupational Harmony

Yijun Liu[1], Ruiqi She[2], and Jin Xing[3]

[1] Department of Rehabilitation Medicine, Peking University First Hospital

[2] Institute for Health Systems Engineering, College of Engineering, Peking University

[3] Department of Rehabilitation Medicine, Beijing Hospital, National Center of Gerontology; Institute of Geriatric Medicine, Chinese Academy of Medical Sciences

Author Note

Yijun Liu https://orcid.org/0000-0002-8983-8576

Jin Xing https://orcid.org/0009-0002-4664-9959

We have no conflicts of interest to disclose. This article is based on the unpublished doctoral dissertation completed by YL. The data used to support the findings of this study are included within the article and supporting information files.

YL thanks Dr. Zhen-Su She for his guidance throughout this work; Dr. Zhen Huang and Dr. Rongli Wang for their mentorship; Dr. Shuna Song, Dr. Rong Li, Dr. Lei Zhang, and her parents for their encouragement and support.

Correspondence concerning this article should be addressed to Yijun Liu, Department of Rehabilitation Medicine, Peking University First Hospital, No. 7 Xishiku Street, Xicheng District, Beijing,100034, P.R. China. Email: yijunliu@pku.edu.cn

ABSTRACT

Background: Mental health problems are common among university students. Based on the Model of Occupational Harmony, we investigated the relationship between occupational engagement and mental health among Chinese university students.

Methods: A mixed methods approach was adopted. A total of 734 Chinese university students responded to online questionnaires: a socio-demographic questionnaire, the Depression Anxiety Stress Scale, the WHOQOL-BREF, and a self-designed Occupational Harmony Questionnaire. Individual interviews with 11 university students and a focus group of four students provided qualitative data.

Results: Anxiety, depression, and stress were reported by 11.9%, 9.4%, and 2.9% of the participants, respectively. The level of occupational harmony was negatively correlated with depression, anxiety, and stress ($p < .001$). The DASS-21 score was significantly different among students who spent different amounts of time on the following activities: exercise, sleep, rest, reflective and consulting activities, study, work, and leisure and entertainment ($P < .05$). In the context of the COVID-19 pandemic, the key occupational factors contributing to university students' mental health problems included excessive mental engagement and insufficient physical engagement, excessive active engagement and insufficient quiet engagement, insufficient social engagement, excessive or insufficient *Gong* (productive) engagement, and insufficient *De* (virtuous) engagement, *Zhi* (rational) engagement, and *Kang* (health maintenance) engagement, lack of coherence within the family, school, social systems, as well as inadequate schedule planning and time management.

Conclusions: Occupational disharmony is related to university students' mental health problems. Occupational therapy can promote university students' mental well-being by enhancing occupational harmony.

Keywords: occupational engagement, occupational harmony, mental health, university students, COVID-19 pandemic

Analysis of University Students' Mental Health from the Perspective of Occupational Harmony

Introduction

University students are a key population segment for determining the economic growth and development of a society. However, they are also at high risk for distress and mental health problems [1]. Globally, the high prevalence of mental disorders among university students has become a significant and growing public health issue [2]. In a survey conducted by the World Health Organization (WHO), 31.4% (95% *CI* 30.2–32.6%) of college students screened positive for at least one common mental disorder [3].

Among the research on university students' mental health, most studies adopted a biomedical perspective using psychiatric symptomatology to investigate the "mental health" status [3-5]. Measurement tools commonly used include the General Anxiety Disorder-7 (GAD-7), the Patient Health Questionnaire (PHQ-9), and the Symptom Checklist-90 (SCL-90), etc., which can identify people who manifest mental illness symptoms. However, it should be noted that mental health is not simply an absence of mental illness. According to the definition by the WHO, mental health refers to "a state of well-being, in which an individual realizes his or her own abilities, can cope with the normal stresses of life, can work productively and is able to make a contribution to his or her community" [6, p. 12].

Two implicit problems exist with using psychiatric symptomatology to study university students' mental health. First, the multifaceted meaning of "mental health" has not been fully explored as it was only used as a euphemism for "mental illness" [7]. Second, as mental health can be seen as a spectrum [8], students with mild

distress but were absent from mental illness symptoms may not be identified. Therefore, it is imperative to investigate mental health from a more positive, functional, and holistic perspective.

The profession of occupational therapy (OT) possesses knowledge and expertise in how people's participation in everyday life shapes their health and well-being [9]. As a central concept in OT, the term "occupational engagement" can offer a functional and performance-based lens to explore mental health in the context of everyday living. Engagement in occupation refers to the "performance of occupations as the result of choice, motivation, and meaning within a supportive context" [10, p. 5], including both objective and subjective aspects of a person’s experience. Previous studies provided evidence that a balance of occupational engagement could be beneficial for university students' mental well-being [11, 12]. In contrast, disruptions to occupational engagement and balance were associated with poor mental health [13].

As cultural values and traditions shape how occupational engagement [14] and mental health (6) are conceptualized across contexts, a Chinese occupational therapy model, the Model of Occupational Harmony (MOHar) [15, 16], was adopted to analyze the mental health of Chinese university students from an occupational lens in this study. The MOHar was built upon traditional Chinese culture and Chinese scholars' Human Complex Systems Theory [17], which provided a conceptual framework for understanding how the orchestration of everyday occupations relates to health and well-being. Notably, the MOHar addressed that occupational engagement was driven by human consciousness [15] and thus assumed an inherent relationship between occupational engagement and mental health.

The term "occupational harmony" indicated harmonious human-environment

transactions through occupational engagement. It was attained when a person achieved their ultimate goal of a satisfactory life [15]. The model proposed that occupational harmony can be characterized as complex equilibria among three pairs of two-sided occupational characteristics (i.e., physical and mental engagement, quiet and active engagement, individual and social engagement), harmony among five-dimensional occupational engagement (i.e., *De* [virtuous] engagement, *Zhi* [rational] engagement, *Gong* [productive] engagement, *Ai* [emotional] engagement, *Kang* [health maintenance] engagement) and coherence across multiple levels (i.e., ontosystem, small-system, medium-system, large-system, chronosystem) of human-environment transactions, which were essential to health and wellbeing.

This study used the MOHar as a theoretical base and had three aims. First, to examine university students' mental health from the perspective of occupational harmony. It was hypothesized that a higher level of occupational harmony would be negatively associated with depression, anxiety, and stress symptoms. Second, to analyze the key occupational factors causing university students' mental distress. It was hypothesized that being over-, under-, or even un-engaged by occupations with a particular characteristic can lead to occupational disharmony and mental health problems. Third, as the MOHar was a theoretical model recently developed, we aimed to generate empirical evidence for the MOHar and explore its fit in analyzing occupational engagement and mental health among university students. We hypothesized that occupational harmony is related to a higher level of mental well-being and quality of life. In contrast, occupational disharmony would result in mental health problems.

Methods

Design

A mixed method approach [18] was chosen to answer the research aims. The MOHar was used both deductively and inductively in collecting and analyzing data. In the quantitative phase, a cross-sectional online survey was conducted to identify the level of occupational harmony and its relationship to mental health and quality of life. In the qualitative phase, individual interviews and a focus group were performed to explore how harmonious occupational engagement affects mental health from the life experience of participants.

Participants

Participants were recruited virtually through the WeChat social media platform. Inclusion criteria included undergraduate or graduate students enrolled in the university at the time of the study who were able to communicate in Mandarin. Students in a state of leave of absence or suspension were excluded. In the quantitative phase, convenience sampling and snowball sampling were used with 734 participants included. In the qualitative phase, participants were selected from the same sample using purposeful sampling and convenience sampling. Eleven students participated in individual interviews and four students participated in a focus group discussion. Ethical approval was obtained from the ethics committee of Peking University First Hospital (Approval number: 2020Study104) and all participants agreed to the written informed consent prior to participation.

Data collection and procedures

All data collection occurred from November 22, 2020 to December 30, 2020.

Quantitative data was collected via a self-report survey through the Baishuyun online data collection platform. A total of 792 survey responses were received. Duplicate responses from the same IP address, respondents who did not meet the inclusion or exclusion criteria, and answers with obvious logic errors were eliminated to ensure the authenticity and reliability of the data. Qualitative data were collected from semi-structured interviews and a focus group discussion conducted virtually via the Tencent video conference. Both visual and audio recordings were obtained. Each individual interview was 40-100 minutes long and the focus group lasted 86 minutes. After the interview, the recordings were transcribed into text data using the Sogo transcription service. Field notes and reflective journals were completed by two researchers (YL and RS).

Instruments and Measures

A structured survey containing informed consent, a socio-demographic questionnaire, the Depression Anxiety Stress Scale (DASS-21), the World Health Organization Quality of Life-BREF (WHOQOL-BREF), and a self-designed Occupational Harmony Questionnaire (OHQ), was used to collect quantitative data.

Socio-Demographic Questionnaire

The socio-demographic questionnaire sought the participants' background information such as gender, age, ethnicity, type of degree, major, monthly family income, and previous mental health issues.

The Depression Anxiety Stress Scale (DASS-21)

The Chinese version of DASS-21 [19] was used to measure symptoms of mental illness. This instrument consists of 21 items including three subscales: 7 items each for depression, anxiety, and stress with a four-point Likert scale ranging from 0 (“never”)

to 3 (“always”) [20]. The total sub-scores range from 0 to 42 and are categorized into normal, mild, moderate, severe, and extremely severe. In this study, Cronbach’s alpha for the depression, anxiety, and stress subscales were 0.89, 0.80, and 0.84, respectively, and the overall DASS-21 scale was found to have good reliability (Cronbach’s alpha = 0.94).

The World Health Organization Quality of Life-BREF (WHOQOL-BREF)

The Chinese version of WHOQOL-BREF [21] was used to assess the quality of life (QoL). This scale contains 26 items assessing QoL in four domains: physical health, psychological health, social relations, and the environment with a five-point Likert scale ranging from 1 to 5. Transformed scores for each domain were from 0 to 100. In this study, the WHOQOL-BREF scale demonstrated good reliability (Cronbach’s alpha = 0.91).

The Occupational Harmony Questionnaire (OHQ)

The OHQ was designed by the research team based on the MOHar to assess the objective and subjective aspects of occupational engagement, respectively. The questionnaire underwent three rounds of revision, including feedback from 10 university students, two occupational therapists, and five university teachers, cognitive interviewing [22] with two university students, and a pilot study conducted with 36 participants.

The objective subscale consists of 17 items to collect the behavior patterns of occupations across the five dimensions. An example of a question is "What is the average time you spend studying every day (including attending classes, conducting research, and internships)?” Participants responded by selecting a time category they fitted into. The subjective subscale consists of 25 items, assessing self-perceived

occupational harmony levels. Participants were asked to respond on a five-point Likert scale (1 = completely inconsistent, 5 = completely consistent) to statements like “Generally speaking, I am very satisfied with my current life." Scores were summed up and converted to a transformed score ranging from 1 to 100. A higher score indicated a higher perceived level of occupational harmony. In this study, the OHQ subjective subscale was found to have good reliability (Cronbach’s alpha = 0.91).

Data Analysis

Quantitative analysis

Of the 792 survey responses, 734 participants were included in the quantitative analysis. The Statistical Package for the Social Sciences (SPSS) (Version 26.0) was used for data analysis. The normality of the data was analyzed using the Shapiro-Wilk test. The demographic data were analyzed using descriptive statistics. Criterion validity of the OHQ subjective subscale was evaluated by the correlation between the level of occupational harmony and quality of life, using the Pearson correlation coefficient. The correlation between the level of occupational harmony and mental health was analyzed using the Spearman correlation coefficient. Comparisons of mental health status among students with different occupational engagement profiles were analyzed using the Kruskal-Wallis *H* test. The Bonferroni method was used for post-hoc analysis. Significance was evaluated at levels of .05.

Qualitative analysis

A combination of deductive and inductive approaches was used in analyzing qualitative data. First, three main themes (i.e., two-sided occupational characteristics, five-dimensional occupational engagement, and multiple levels of human-environment

transactions) and subthemes were developed deductively from the MOHar (See the S1 Table for details). Then, meaningful excerpts from the data were assigned to the predetermined themes. New codes describing mental health status (e.g., anxious, stressed, depressive) also emerged from the data. Based on the above analysis, a profile of each participant's occupational engagement and mental health status was developed and the relationship between occupational engagement and mental health was further analyzed using the MOHar. Next, similarities and differences in participants' occupational engagement and mental health status were identified by comparing all profiles, generating common occupational factors contributing to mental health problems. All analyses were conducted by two researchers (YL and RS) with multiple discussions. The quotes were translated into English using forward translation.

Results

Characteristics of Participants

Among the 734 participants, 518 (70.6%) were females and 576 (78.4%) were aged 19-24 years. Fifteen of them participated in the interviews and a focus group discussion. Table 1 and Table 2 show participant characteristics in the quantitative and qualitative phase of this study, respectively. Detailed profiles of 15 participants in the qualitative study were included in the S2 Table.

Table 1. Participant Characteristics in the Quantitative Study (N=734)

Characteristics	No. (%)
Gender	
Male	216 (29.4)
Female	518 (70.6)
Age (years)	
≤18	58 (7.9)
19-21	371 (50.5)

22-24	205 (27.9)
25-27	61 (8.3)
≥28	39 (5.3)
Ethnicity	
Han	689 (93.9)
Minorities	45 (6.1)
Types of degree	
Undergraduate	493 (67.2)
Graduate	241 (32.8)
Major	
Medical	223 (30.4)
Non-medical	511 (69.6)
Location of university	
Mainland China	705 (96.0)
Out of China	29 (4.0)
Monthly family income (Yuan)	
＜1000	48 (6.5)
1001-3000	231 (31.5)
3001-5000	172 (23.4)
5001-7000	115 (15.7)
7001-10000	79 (10.8)
≥10001	89 (12.1)
Prior mental health issues	
Yes	29 (4.0)
No	705 (96.0)

Table 2. Participant Characteristics in the Qualitative Study (N=15)

Characteristics	Median	Range
Age	21	19-28
OHQ subjective subscale score	70.4	47.2-79.2
Characteristics	No.	Percentage
DASS-21 score		
Positive	5	33.3%

Negative	10	66.7%
Gender		
Male	3	20.0%
Female	12	80.0%
Types of degree		
Undergraduate	10	66.7%
Graduate	5	33.3%
Prior mental health issues		
Yes	2	13.3%
No	13	86.7%

OHQ: The Occupational Harmony Questionnaire, DASS-21: The Depression Anxiety Stress Scale.

Quantitative Results

Occupational Engagement Status

In the OHQ objective subscale, we investigated university students' engagement in 10 occupations across five dimensions. Fig 1 shows the percentage of participants' average time-use in five-dimensional occupational engagement. The median score of the OHQ subjective subscale was 72.8 (IQR 64.0-79.2).

Fig 1. Percentage of Participants' Average Time-Use in Five-Dimensional Occupational Engagement (N=734)

<insert Fig 1 here>

Occupational Harmony and Quality of Life

The level of occupational harmony demonstrated significant positive correlations with the four domains of QoL: physical health (r=0.618, $P < .001$), psychological health (r=0.668, $P < .001$), social relations (r=0.512, $P < .001$), and environment

(r=0.503, $P < .001$), showing occupational harmony is positively related to the quality of life and the OHQ subjective scale has a good criterion validity.

Occupational Harmony and Mental Health

Among the 734 participants, 116 (15.8%) had depressive, anxiety, or stress symptoms. The prevalence rates were 9.4%, 11.8%, and 2.8%, respectively. The level of occupational harmony showed significant negative correlations with depression (r=-0.628, $P < .001$), anxiety (r=-0.500, $P < .001$), and stress (r=-0.553, $P < .001$). Table 3 shows the relationships between OHQ subjective subscale scores and DASS-21 scores.

Table 3. Relationship between OHQ Subjective Subscale Scores and DASS-21 Scores (N=734)

	Depression		Anxiety		Stress	
	r	*P value*	r	*P value*	r	*P value*
Level of occupational harmony	-.628	<.001	-.500	<.001	-.553	<.001
Equilibrium between two-sided occupational characteristics	-.459	<.001	-.395	<.001	-.465	<.001
Physical and mental engagement	-.398	<.001	-.331	<.001	-.397	<.001
Quiet and active engagement	-.404	<.001	-.364	<.001	-.425	<.001
Individual and social engagement	-.410	<.001	-.333	<.001	-.397	<.001
Five-dimensional occupational engagement	-.624	<.001	-.482	<.001	-.525	<.001
De engagement	-.453	<.001	-.351	<.001	-.377	<.001
Zhi engagement	-.435	<.001	-.342	<.001	-.333	<.001
Gong engagement	-.490	<.001	-.367	<.001	-.390	<.001
Ai engagement	-.439	<.001	-.338	<.001	-.359	<.001
Kang engagement	-.599	<.001	-.473	<.001	-.554	<.001
Multiple levels of human-environment transactions	-.516	<.001	-.398	<.001	-.449	<.001
Small-system	-.349	<.001	-.312	<.001	-.339	<.001
Medium-system	-.260	<.001	-.209	<.001	-.213	<.001
Large-system	-.341	<.001	-.239	<.001	-.301	<.001
Chronosystem	-.426	<.001	-.315	<.001	-.331	<.001

OHQ: The Occupational Harmony Questionnaire, DASS-21: The Depression Anxiety Stress Scale.

Occupational Factors Associated with Mental Health Problems

Participants who studied less than 2 hours a day, slept less than 6 hours a day, rested less than 40 minutes per week, or had unhealthy eating patterns, demonstrated higher scores for depression, anxiety, and stress than the other groups. Participants

who studied or worked more than 10 hours a day had a higher score for anxiety. Participants who did not participate in any reflective and consulting activities had a higher score for depression. Participants who exercised over 150 minutes per week had a lower score for depression. Students spending time on leisure and entertainment for less than 1 hour had lower scores for depression, anxiety, and stress than the other groups. Table 4 shows the difference in DASS-21 scores of university students with different time-use in various occupations.

Table 4. Differences in DASS-21 Scores of University Students with Different Time-Use in Five-Dimensional Occupational Engagement (N=734)

Occupational engagement	Depression subscale score		Anxiety subscale score		Stress subscale score	
	M (P25，P75)	*P* value	M (P25，P75)	*P* value	M (P25，P75)	*P* value
Collective activities (*De* engagement)		.154		.749		.145
0 h/d	3.0 (2.0，7.0)		4.0 (2.0，6.0)		6.0 (3.0，9.0)	
＜1 h/d	3.0 (1.0，6.0)		3.0 (2.0，6.0)		6.0 (3.0，7.0)	
1-2 h/d	3.0 (1.0，6.0)		3.0 (2.0，6.0)		6.0 (3.0，7.5)	
2-3 h/d	4.0 (3.0，7.0)		5.0 (2.0，7.0)		7.0 (3.0，8.0)	
≥3 h/d	2.0 (0.0，7.0)		3.0 (1.0，6.5)		4.0 (0.0，7.0)	
Reflective and consulting activities (*Zhi* engagement)		.002		.650		.260
0 h/d	4.5 (2.0，7.0)		4.0 (2.0，6.0)		6.0 (4.0，8.0)	
＜0.5 h/d	3.0 (1.0，7.0)		3.0 (2.0，6.0)		6.0 (3.0，8.0)	
0.5-1 h/d	3.0 (1.0，6.0)		4.0 (2.0，6.0)		6.0 (3.0，7.0)	
1-2 h/d	3.0 (0.0，4.0)		3.0 (1.0，5.8)		5.0 (2.0，8.0)	
≥2 h/d	3.0 (1.0，7.5)		3.0 (1.0，7.0)		5.0 (3.0，8.0)	
Study (*Gong* engagement)		＜.001		＜.001		＜.001
＜2 h/d	7.0 (2.3，11.0)		5.5 (3.0，9.0)		8.0 (5.0，11.0)	
2-4 h/d	5.0 (1.0，7.0)		4.0 (2.0，7.0)		6.0 (2.0，8.0)	
4-6 h/d	3.0 (1.0，6.0)		3.0 (2.0，6.0)		5.0 (3.0，7.0)	

6-8 h/d	3.0 (1.0，6.0)		3.0 (2.0，5.0)		5.0 (3.0，7.0)	
8-10 h/d	3.0 (1.0，5.0)		2.0 (1.0，5.0)		5.0 (2.0，7.0)	
≥10 h/d	4.0 (2.0，7.0)		4.0 (3.0，7.0)		7.0 (4.0，9.8)	
Work (*Gong* engagement)		.814		.049		.317
＜2 h/d	3.0 (1.0，7.0)		3.0 (2.0，6.0)		6.0 (3.0，8.0)	
2-4 h/d	3.0 (1.0，7.0)		4.0 (1.8，6.0)		6.0 (2.0，7.0)	
4-6 h/d	4.0 (1.0，6.0)		4.0 (2.0，6.0)		5.0 (2.0，7.8)	
6-8 h/d	3.0 (0.0，7.0)		3.0 (1.0，6.0)		6.0 (3.0，9.0)	
8-10 h/d	3.0 (0.8，6.0)		2.0 (1.0，3.3)		4.5 (2.8，6.3)	
≥10 h/d	3.5 (2.0，7.0)		5.5 (3.0，9.0)		7.5 (4.0，9.8)	
Leisure and entertainment (*Ai* engagement)		＜.001		.003		.010
0 h/d	5.0 (2.0，8.0)		4.0 (2.0，7.0)		7.0 (4.0，10.0)	
＜1 h/d	3.0 (1.0，5.0)		3.0 (1.0，5.0)		5.0 (3.0，7.0)	
1-2 h/d	3.0 (1.0，6.0)		4.0 (2.0，6.0)		6.0 (3.0，7.0)	
2-3 h/d	4.0 (2.0，7.0)		4.0 (2.0，6.0)		6.0 (3.0，8.0)	
3-4 h/d	4.5 (2.0，8.0)		4.0 (2.0，7.0)		7.0 (3.0，8.3)	
≥4 h/d	5.5 (2.0，9.0)		4.0 (1.0，6.3)		7.0 (3.0，10.3)	
Social activities (*Ai* engagement)		.404		.126		.351
0 h/d	5.0 (0.0，7.0)		3.0 (2.0，6.0)		5.0 (4.0，8.0)	
＜1 h/d	3.0 (1.0，7.0)		3.0 (1.0，6.0)		6.0 (3.0，7.0)	

1-2 h/d	3.0 (1.8，6.0)		3.0 (2.0，6.0)		6.0 (3.0，8.0)	
2-3 h/d	4.0 (2.0，7.0)		4.0 (2.0，6.0)		7.0 (3.5，8.0)	
3-4 h/d	3.0 (0.0，7.8)		3.0 (1.0，7.0)		5.5 (3.0，8.8)	
≥4 h/d	4.0 (3.0，6.0)		5.0 (3.0，6.0)		6.0 (3.0，7.0)	
Sleep (*Kang* engagement)		.006		＜.001		＜.001
＜6 h/d	6.0 (3.0，8.0)		6.0 (3.0，8.0)		7.0 (5.0，9.0)	
6-7 h/d	3.0 (1.0，6.0)		4.0 (2.0，6.0)		6.0 (3.0，8.0)	
7-8 h/d	3.0 (1.0，6.0)		3.0 (1.0，5.0)		5.0 (3.0，7.0)	
8-9 h/d	3.0 (1.0，6.0)		3.0 (1.0，6.0)		5.0 (2.0，8.0)	
≥9 h/d	4.0 (1.5，9.3)		2.5 (1.0，8.8)		4.5 (2.8，12.3)	
Rest (*Kang* engagement)		＜.001		.003		＜.001
0-40 min/w	4.0 (2.0，8.0)		4.0 (2.0，7.0)		7.0 (4.0，9.0)	
40-100 min/w	3.0 (1.0，6.0)		4.0 (2.0，6.0)		6.0 (3.0，8.0)	
100-200 min/w	3.0 (1.0，6.0)		3.0 (2.0，5.0)		5.0 (3.0，7.0)	
≥200 min/w	2.0 (1.0，6.0)		3.0 (1.0，5.0)		5.0 (2.0，7.0)	
Diet (*Kang* engagement)		＜.001		＜.001		＜.001
Regular and healthy	2.0 (0.0，4.0)		2.0 (1.0，4.0)		4.0 (2.0，6.0)	
Healthy but irregular	4.0 (2.0，7.0)		4.0 (2.0，7.0)		6.0 (3.0，8.0)	
Regular but unhealthy	3.0 (2.0，7.0)		4.0 (2.0，6.0)		6.0 (4.0，8.0)	
Irregular and unhealthy	6.0 (3.0，9.0)		5.0 (3.0，7.8)		7.0 (5.0，10.0)	
Exercise (*Kang* engagement)		.039		.011		.002

0 min/w	4.0 (2.0，7.0)	4.0 (2.0，6.0)	6.0 (4.0，8.0)
0-149 min/w	3.0 (1.0，6.5)	4.0 (2.0，6.0)	6.0 (3.0，8.0)
≥150 min/w	3.0 (0.0，6.0)	3.0 (1.0，5.0)	4.0 (2.0，7.0)

DASS-21: The Depression Anxiety Stress Scale.

Qualitative Findings

Participants' occupational engagement and mental health status were analyzed according to the MOHar. Three main themes and their subthemes were used to guide data analysis as follows.

Theme 1: Two-Sided Occupational Characteristics and Mental Health

The MOHar proposed that equilibria between three pairs of two-sided occupational characteristics were essential for health and well-being [15]. The state of equilibrium is determined by the individual's self-perception and favorable time allocation. In this study, we found that excessive mental and active engagement and insufficient social engagement were common among university students, which contributed to mental health problems.

Subtheme 1.1: Disequilibrium Between Mental and Physical Engagement. Under the influence of COVID-19, excessive screen time and limited physical exercise caused an imbalance between mental and physical engagement. Three participants reported they were addicted to the Internet during the home isolation period, making them "feel guilty about wasting time". Four participants spoke about "feeling a lack of exercise" due to excessive workload or the home quarantine policy. For example, participant F stated that "I was reading the literature every day, which made me become silly... I was afraid that my body could not endure this".

Subtheme 1.2: Disequilibrium Between Active and Quiet Engagement. Excessive engagement in the study or entertainment and lack of sleep were common problems identified by the participants. Five participants reported, "staying up late had become a habit" and "often went to bed at 3-4 AM". Some of them stayed up late

to study, which made them feel "irritable", "anxious" and even "despairing". Others spent the night watching TV shows or reading novels, which made them lack the energy and motivation to study and work and caused depressive and anxiety symptoms.

Subtheme 1.3: Disequilibrium Between Individual and Social Engagement. Due to the COVID-19 protective measures, many participants reported a lack of social engagement, making them feel "bored", "lonely", and even "annoyed". Six participants complained about not being able to hang out or travel. For example, participant B said, "I was annoyed about being confined at home and unable to participate in other activities and meet other people". Five participants reported "feeling lonely" as they were very eager to communicate with their peers.

Theme 2: Five-Dimensional Occupational Engagement and Mental Health

In the MOHar, it was asserted that occupational harmony, or a satisfactory life, was achieved through harmonious engagement in the five dimensions of occupations [15]. The five dimensions of occupational engagement have mutually enhancing and restricting relationships. The collective compatibility of the configuration of occupations is essential for mental health.

Among the 15 participants, 12 of them expressed dissatisfaction with their current life. Three types of common occupational disharmony patterns among participants were identified as shown in Fig 2.

Fig 2. Typical Occupational Disharmony Patterns of Participants (N=15). The size of Taiji diagrams indicates the level of occupational engagement. The Taiji diagram in

the middle represents a moderate degree of occupational engagement. A smaller size indicates insufficient engagement, while a larger size indicates excessive engagement.

<insert Fig 2 here>

Subtheme 2.1: Excessive *Gong* Engagement and Insufficient *Kang* Engagement. Five participants demonstrated excessive *Gong* engagement, which directly restricted their *Kang* engagement (Pattern A in Fig 2). Specifically, these students were under great pressure to complete their coursework or research projects. Their daily life was very busy and they often "stayed up late to work on assignments”. They lacked sleep and didn't have enough time for rest and exercise. Under excessive workload, these students were more likely to feel stressed, irritable, and even despairing.

Subtheme 2.2: Insufficient *Zhi* and *Gong* Engagement. Five participants demonstrated insufficient *Zhi* engagement, which also negatively impacted their *Gong* engagement (Pattern B). The main problem with this group of students was that they had challenges in their studies that were difficult to solve by themselves. However, they seldom actively find solutions or consult with others, resulting in poor grades or slow research progress. The "self-isolated" status along with academic pressure caused depression, stress, and anxiety symptoms.

Subtheme 2.3: Insufficient *De*, *Zhi*, and *Gong* Engagement. Two participants showed insufficient engagement in the *De* engagement, which negatively impacted their *Zhi* and *Gong* engagement (Pattern C). These students were not clear about their future goals and plans. They had difficulty with making choices and did not know where to put effort. They were not able to fully engage in their studies and work and thus lacked academic achievement, which made them feel confused and anxious about their future.

Theme 3: Multiple Levels of Human-Environment Transactions and Mental Health

The essence of occupational harmony was proposed to be harmonious human-environment transactions across multiple levels [15]. Four levels of systems were analyzed, which were small-system, medium-system, large-system, and chronosystem.

Subtheme 3.1: Coherence with the Small-System. As most students stayed at home during the pandemic, the small system was usually composed of their home and family members. We found that participants' relationships with family members had a big impact on their moods. Five participants reported having arguments or conflicts with their parents, which made them feel displeased. Participant G even ran away from home because she couldn't tolerate her mother. In contrast, three participants had a more regular and enjoyable life at home than at school because of their good relationships with their families.

Subtheme 3.2: Coherence with the Medium-System. For university students, the medium system mainly involves their schools. During the quarantine period, many students had to leave their schools and study on online learning platforms at home. Eight participants reported dissatisfaction with online learning because it was difficult to concentrate and communicate with teachers and classmates. The lack of a learning atmosphere made them "lack the motivation to learn and often slack off when taking online classes". Conversely, eight participants mentioned they felt stressed at school due to academic pressure.

Subtheme 3.3: Coherence with the Large-System. Large systems include societies, countries, and the world. Due to the global pandemic, three participants'

study abroad and exchange plans were disrupted. For example, student I had prepared for studying in Japan for a long time, but the outbreak of the pandemic "completely disrupted the original plan". When she recalled the experience, she said "I was so devastated...It was extremely hard for me to accept this fact...This was the biggest impact that the pandemic had on me."

Subtheme 3.4: Coherence with the Chronosystem. Coherence with the chronosystem mainly refers to sufficient planning and management of time. Eight participants reported having no clear goals for life or lacking reasonable planning. Three participants felt that life was "boring" and "meaningless" and did nothing every day. Student J is a fifth-year doctoral student who has been postponed for two years due to mental illness. Although he was "eager to graduate next year", he did not have a plan for it and was also anxious and worried about meeting the graduation requirements.

Discussion

This study investigated university students' mental health from the perspective of occupational harmony. The quantitative and qualitative results verified the primary hypotheses proposed by the MOHar. The results of the correlation analyses showed that the level of occupational harmony was positively correlated with QoL and negatively correlated with depression, anxiety, and stress, indicating that occupational harmony can lead to higher QoL and better mental health.

Although mental illness symptoms vary and the influencing factors are complex, the MOHar provides a theoretical framework to systematically analyze how occupational engagement impacts mental health. More than identifying the association relationships, we attempted to explain the causal relationships between

occupational engagement and mental health based on the theoretical propositions of the MOHar.

According to the MOHar, it is believed that maintaining moderate engagement in occupations with different characteristics is essential for health and well-being. Disequilibrium between the two-sided (*Yin* and *Yang*) characteristics of occupational engagement or disharmony among the five-dimensional occupational engagement will lead to mental illness [15]. The integration of quantitative and qualitative results indicates that students over-, under-, or even un-engaged in particular occupations are more likely to have mental illness symptoms, whereas moderate engagement is related to better mental health.

In terms of two-sided occupational characteristics, qualitative findings show that excessive mental engagement (e.g., excessive screen time) and insufficient physical engagement (e.g., lack of physical exercise), excessive active engagement (e.g., excessive study or entertainment) and insufficient quiet engagement (e.g., lack of sleep or rest), as well as insufficient social engagement (e.g., limited social activities), are related to mental health problems. There are studies that similarly found that the absence of physical exercise [23], heavy internet use (≥5 h/d) [24], poor sleep [25], and increased social isolation [26] were associated with stress, anxiety, and depression symptoms.

Regarding the five-dimensional occupational engagement, we found three common occupational disharmony patterns among university students, which were also supported by quantitative findings. The first pattern is characterized by excessive *Gong* engagement and insufficient *Kang* engagement, causing stress and anxiety symptoms. The Kruskal-Wallis *H* test shows that excessive *Gong* engagement (i.e., study more than 10h/d) is related to an increased level of anxiety, while insufficient

Kang engagement (i.e., sleep less than 6h/d, rest less than 40min/w, unhealthy eating) is related to higher levels of depression, anxiety, and stress symptoms. The second pattern is characterized by insufficient *Zhi* and *Gong* engagement, causing depression, stress, and anxiety symptoms. Quantitative results indicate that insufficient *Zhi* engagement (i.e., reflective and consulting activities) contributed to a higher level of depression, and insufficient *Gong* engagement (i.e., studied less than 2h/d) is related to higher levels of depression, anxiety, and stress symptoms. The third pattern is characterized by insufficient *De*, *Zhi*, and *Gong* engagement, similar to the second pattern. Due to the representative activity for *De* engagement (i.e., collective activity) being different from the activities participants reported in the interview, we are not able to integrate the quantitative and qualitative findings for this pattern here. Future studies can further verify the relationship between life-planning activities and mental health.

Part of the above findings are consistent with previous literature. For instance, scholars found that difficulty with concentrating on academic work (*Gong* dimension) [26], disruptions to sleep and eating patterns (*Kang* dimension) [26] and lack of exercise (*Kang* dimension) [23], contributed to the increased levels of stress, anxiety, and depressive symptoms among students during the COVID-19 pandemic. To our knowledge, occupations in *De* and *Zhi* dimensions were seldom examined in previous studies, partially due to differences in cultural values and traditions. Additionally, the interrelationships among the five dimensions of occupation were revealed in the qualitative findings. For example, excessive *Gong* engagement can inhibit *Kang* engagement. Insufficient *De* engagement can lead to insufficient *Zhi* and *Gong* engagement. Analysis of these interrelationships is important for identifying key factors and thus informing intervention planning.

As Chinese culture is highly collectivist with a strong emphasis on group belonging and harmony [27], coherence across multiple levels of human-environment transactions is believed essential for mental well-being. Incoherence with the family (i.e., conflicts with family members), school (i.e., challenges of online learning, competitive pressure), social systems (i.e., maladaptation to COVID-19 measures), and the chronosystem (i.e., poor time management) were identified. Previous research also found that changes in the living environment [26] and poor family relationships [28] had an adverse effect on mental disorder symptoms. However, a paucity of studies explored environments from a multi-level lens and investigated how social systems impact an individual's occupational engagement and mental health. Our findings reveal the mechanisms of how systems at different levels interact with each other and have a complex influence on an individual's mental health.

It is notable that according to the DASS-21 scores, 84.2% of the participants in this study were in normal mental health status. However, their OHQ results indicated that many university students with normal DASS-21 scores still had problems with occupational engagement. This is also supported by qualitative findings. Among the 15 participants, although the DASS-21 scores of 10 participants were within the normal range, they still had problems in different aspects of their life, such as study, career, family, and interpersonal relationships. These results suggest that the MOHar can provide a more holistic and functional perspective to studying university students' mental health.

Some limitations of this study should be acknowledged. Firstly, the study was conducted during the COVID-19 pandemic, which may limit the external validity of the results. Secondly, the survey was based on a convenience sample of university students with 70% female participants, which might result in selection bias and

limited generalizability. Thirdly, to optimize the trade-off between a large number of everyday occupations and the limitation of questionnaire length, we selected some specific activities to represent five-dimensional occupational engagement. As these activities were proposed based on Chinese university students' life experiences and the study was conducted among Chinese students, the scope of transferability might be limited. Fourthly, the self-designed OHQ is not a standardized scale and its psychometric properties should be further tested.

Overall, the relationship between occupational harmony and mental health verified in this study provides a foundation for developing OT interventions. Future research can focus on developing interventions based on the MOHar to promote university students' mental health and test the efficacy and effectiveness of the intervention to bring about desired outcomes and explore the mechanisms that account for changes. This work, in turn, can lead to further theory development as it brings together research observations with interpretive reasoning proposed in the MOHar.

Conclusion

This study investigated university students' occupational engagement and mental health through the lens of the Model of Occupational Harmony. The findings suggested that the level of occupational harmony is positively associated with university students' mental health. Under the COVID-19 pandemic, the key occupational factors affecting university students' mental health included excessive mental engagement and insufficient physical engagement, excessive active engagement and insufficient quiet engagement, insufficient social engagement, excessive or insufficient *Gong* (productive) engagement, insufficient *De* (virtuous) engagement, *Zhi* (rational) engagement, and *Kang* (health maintenance) engagement,

as well as incoherence with the family, school, social systems, and the chronosystem. Occupational therapy enhancing occupational harmony may be a promising treatment option to promote university students' mental health. Future studies could explore the efficacy and effectiveness of intervention programs based on the MOHar.

References

1. Duffy A, Saunders KEA, Malhi GS, Patten S, Cipriani A, McNevin SH, et al. Mental health care for university students: a way forward? Lancet Psychiatry. 2019;6(11):885-887. doi: 10.1016/S2215-0366(19)30275-5.

2. Bantjes J, Hunt X, Stein DJ. Public Health Approaches to Promoting University Students' Mental Health: A Global Perspective. Curr Psychiatry Rep. 2022;24(12):809-818. doi:10.1007/s11920-022-01387-4

3. Auerbach RP, Mortier P, Bruffaerts R, Alonso J, Benjet C, Cuijpers P, et al. WHO World Mental Health Surveys International College Student Project: Prevalence and distribution of mental disorders. J Abnorm Psychol. 2018;127(7):623-638. doi:10.1037/abn0000362

4. Guo LP, Li ZH, Chen TL, Liu GH, Fan HY, Yang KH. The prevalence of mental distress and association with social changes among postgraduate students in China: a cross-temporal meta-analysis. Public Health. 2020;186:178-184. doi:10.1016/j.puhe.2020.06.014

5. Wang X, Hegde S, Son C, Keller B, Smith A, Sasangohar F. Investigating Mental Health of US College Students During the COVID-19 Pandemic: Cross-Sectional Survey Study. J Med Internet Res. 2020;22(9):e22817. Published 2020 Sep 17. doi:10.2196/22817

6. World Health Organization. Promoting mental health: Concepts, emerging evidence, practice (Summary Report). Geneva: World Health Organization; 2004[cited 2024 July 29]. Available from: https://www.who.int/publications/i/item/9241562943

7. Manwell LA, Barbic SP, Roberts K, Durisko Z, Lee C, Ware E, McKenzie K. What is mental health? Evidence towards a new definition from a mixed methods multidisciplinary international survey. BMJ Open. 2015;5(6):e007079. doi: 10.1136/bmjopen-2014-007079.

8. Frank E, Cassano G, Shear MK, Rotondo A, Dell'Osso L, Mauri M, Maser J, & Grochocinski V. The spectrum model: A more coherent approach to the complexity of psychiatric symptomatology. CNS Spectrums. 1998;3(4), 23-34. doi:10.1017/S1092852900005836.

9. World Federation of Occupational Therapists. Definition of Occupational Therapy. 2012[cited 2024 July 29]. Available from: https://wfot.org/resources/definitions-of-occupational-therapy-from-member-organisations

10. American Occupational Therapy Association. Occupational therapy practice framework: Domain and process (4th ed.). Am J Occup Ther. 2020; 74(Suppl. 2), 7412410010. doi:10.5014/ajot.2020.74S2001

11. Pekçetin S, Günal A. Effect of Web-Based Time-Use Intervention on Occupational Balance During the Covid-19 Pandemic. Can J Occup Ther. 2021;88(1):83-90. doi:10.1177/0008417421994967

12. Tapia V, Isralowitz EB, Deng K, Nguyen NT, Young M, Como DH, et al. Exploratory analysis of college students' occupational engagement during COVID-19. J Occup Sci. 2022; 29(4), 545-561. doi:10.1080/14427591.2022.2101021

13. Salar S, Pekçetin S, Günal A, Akel, BS. Time-use, occupational balance, and temporal life satisfaction of university students in Turkey during isolation period of COVID-19. J Occup Sci. 2022; 29(3), 284-294. doi:10.1080/14427591.2022.2031260

14. Hammell KW. Sacred texts: a sceptical exploration of the assumptions underpinning theories of occupation. Can J Occup Ther. 2009;76(1):6-22. doi:10.1177/000841740907600105

15. Liu Y, Zemke R, Liang L, Gray JM. Occupational harmony: Embracing the complexity of occupational balance. J Occup Sci. 2021; 30(2), 145-159. doi:10.1080/14427591.2021.1881592

16. Liu Y, Zemke R, Liang L, Gray JM. The Model of Occupational Harmony: A Chinese perspective on occupational balance. Ann Int Occup Ther. 2021; 4(4), e228–e235. doi:10.3928/24761222-20210601-08

17. She, Z.-S., & Ni, Z. Renti fuza xitong kexue tansuo [An exploration of the complex systems science of human body]. Chinese Science Publishing & Media Ltd; 2012.

18. Creswell JW, Clark VLP. Designing and conducting mixed methods research (3rd ed.). SAGE Publications, Inc; 2018.

19. Gong X, Xie X, Xu R, Luo Y. Psychometric properties of the Chinese versions of DASS-21 in Chinese college students. Chinese Journal of Clinical Psychology. 2010;18(4), 443–446.

20. Antony MM, Bieling PJ, Cox BJ, Enns MW, Swinson RP. Psychometric properties of the 42-item and 21-item versions of the Depression Anxiety Stress Scales in clinical groups and a community sample. Psychol Assessment. 1998;10(2), 176-181. doi:10.1037/1040-3590.10.2.176

21. World Health Organization. The World Health Organization Quality of Life (WHOQOL)-BREF. Geneva: World Health Organization; 2004 [cited 2024 July 29]. Available from: https://www.who.int/tools/whoqol/whoqol-bref/docs/default-source/publishing-policies/whoqol-bref/chinese-whoqol-bref

22. Fowler F. Improving survey questions: Design and evaluation (1st ed.). SAGE Publications, Inc; 1995.

23. Khan AH, Sultana MS, Hossain S, Hasan MT, Ahmed HU, Sikder MT. The impact of COVID-19 pandemic on mental health & wellbeing among home-quarantined Bangladeshi students: A cross-sectional pilot study. J Affect Disord. 2020;277:121-128. doi:10.1016/j.jad.2020.07.135

24. Islam MS, Sujan MSH, Tasnim R, Sikder MT, Potenza MN, van Os J. Psychological responses during the COVID-19 outbreak among university students in Bangladesh. PLoS One. 2020;15(12):e0245083. doi:10.1371/journal.pone.0245083

25. Cellini N, Canale N, Mioni G, Costa S. Changes in sleep pattern, sense of time and digital media use during COVID-19 lockdown in Italy. J Sleep Res. 2020;29(4):e13074. doi:10.1111/jsr.13074

26. Son C, Hegde S, Smith A, Wang X, Sasangohar F. Effects of COVID-19 on college students' mental health in the united states: Interview survey study. J Med Internet Res. 2020;22(9):e21279. doi:10.2196/21279

27. Wei X, Li Q. The Confucian value of harmony and its influence on Chinese social interaction. Cross-Cult Commun. 2013; 9(1), 60-66. doi:10.3968/j.ccc.1923670020130901.12018

28. Chen B, Sun J, Feng Y. How Have COVID-19 Isolation Policies Affected Young People's Mental Health? - Evidence From Chinese College Students. Front Psychol.

2020;11:1529. doi:10.3389/fpsyg.2020.01529

Supporting Information

S1 Table. Themes and Subthemes Proposed by the Model of Occupational Harmony. The two-sided, five-dimensional, and multi-level characteristics can be used to portray each occupational engagement and are not mutually exclusive. The characteristics are mainly determined by people's motivation of and subjective feelings about their engagement (i.e., characteristics of human consciousness that drive occupational engagement).

S2 Table. Profiles of 15 Participants in the Qualitative Study. OHQ: The Occupational Harmony Questionnaire, DASS-21: The Depression Anxiety Stress Scale. Participant experience content areas. a: Characteristics of occupational engagement; b: Overall statement on mental health. Participants A to K participated in individual interviews. Participants L to O participated in a focus group discussion.

Click here to access/download;Figure;Fig 1.tif

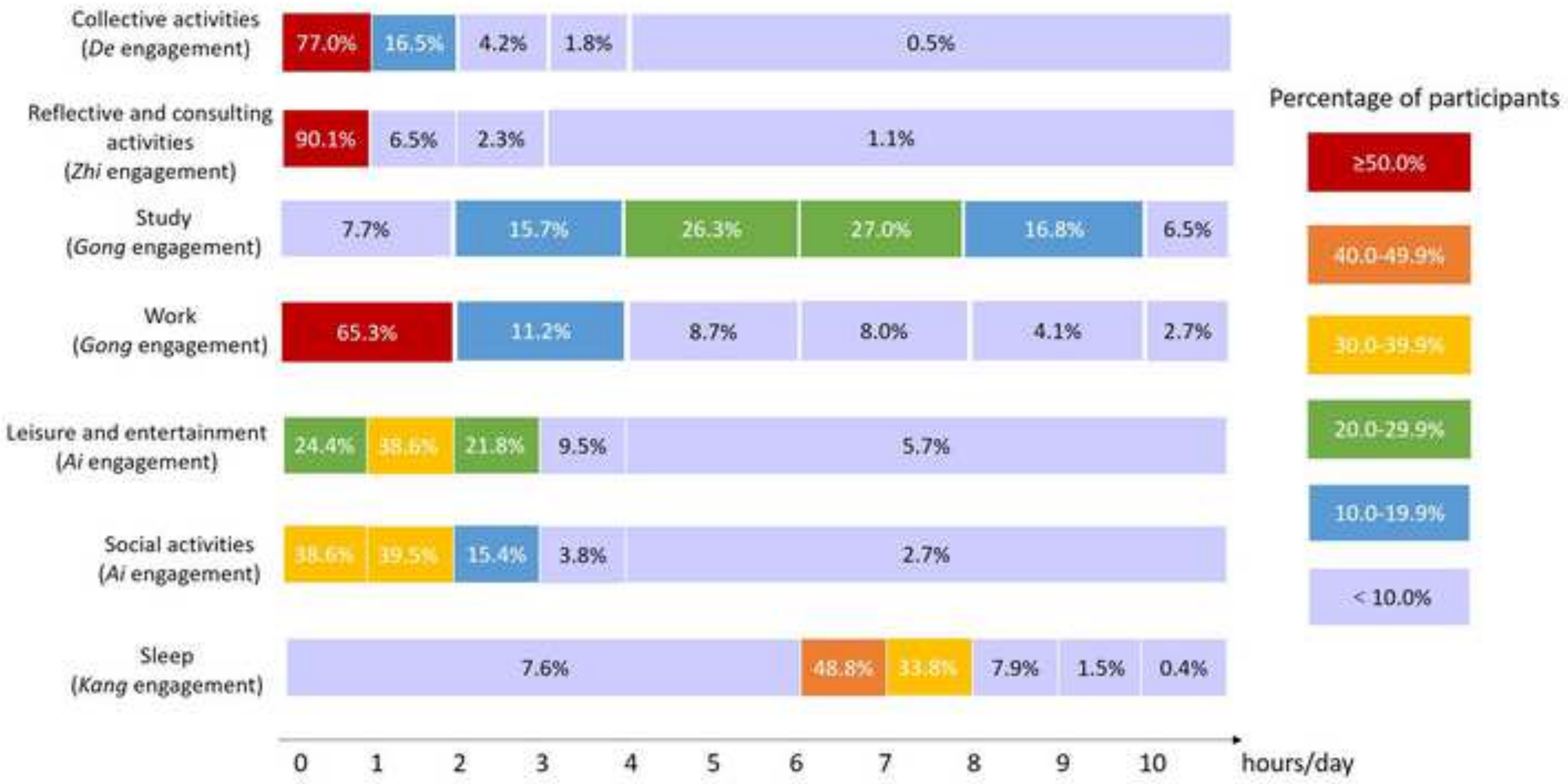

Figure
Click here to access/download;Figure;Fig 2.tif

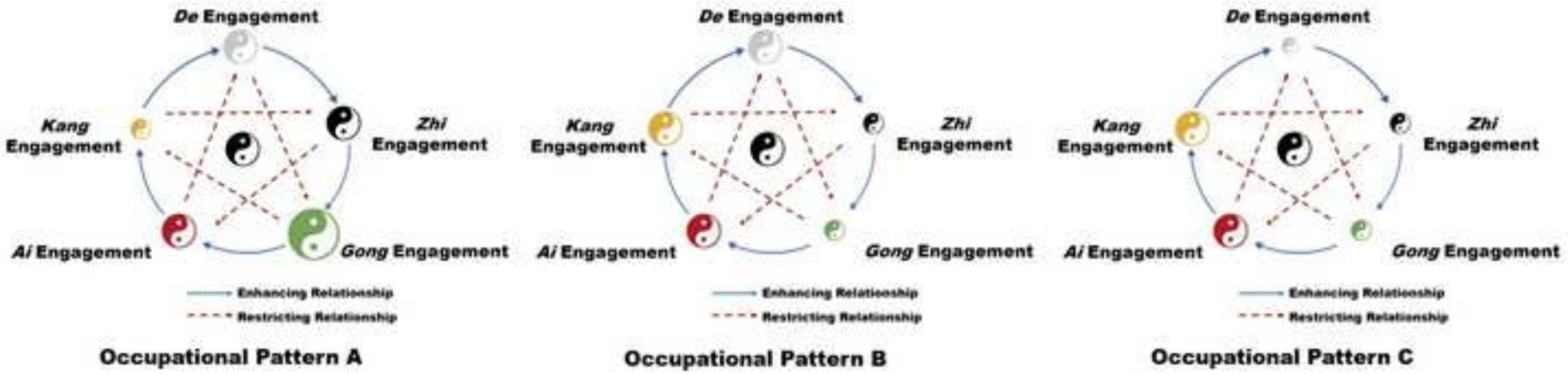

Click here to access/download
Supporting Information
Supporting Information.docx
