# Supplementary material for: Analysis of University Students’ Mental Health from the Perspective of Occupational Harmony
Source: PLoS One. 2025 Apr 1;20(4):e0309490. doi: 10.1371/journal.pone.0309490 (PMC11960997; doi:10.1371/journal.pone.0309490)
Supplement: Attachment — Submitted filename: Response to Reviewers.docx [file pone.0309490.s005.docx]

**Response to Reviewers**

We sincerely thank the academic editor and reviewers for their constructive feedback and valuable comments, which were of great help in revising the manuscript. Accordingly, the manuscript has been systematically revised and improved per their comments. Our responses to the reviewers’ comments are given below. All page numbers refer to the revised manuscript file with tracked changes.

**Reviewers’ Comments to the Authors:**

**Reviewer 1**

- **Comment 1:** Add CONSORT flow chart for participants selection
- **Author Response 1:** Thank you for this suggestion. We have added a flow chart of study population selection in the Participants section on page 7 lines 148-150.
- **Comment 2:** Kruskal Wallis H test-Table for the same
- **Author Response 2:** Table 4 on page 16 lines 291-292 and page 17-20 lines 293-294 demonstrates the results of Kruskal Wallis H test.
- **Comment 3:** Meaningful excerpts- vocabulary correction
- **Author Response 3:** As per the reviewer’s comments, we have changed the phrase “meaningful excerpts” to “meaningful quotes” on page 11 line 228.
- **Comment 4:** Suggest the clinical significance of conducting this study and future scope for the same in the Conclusion section.
- **Author Response 4:** As per the reviewer’s comments, we have added discussion of the clinical significance and future scope of conducting this study on page 29-30 lines 512-532.
- **Comment 5:** Attach the file of Ethical Committee Certificate
- **Author Response 5:** The file of Ethical Committee Certificate both in Chinese and English have been uploaded as attachments.
- **Comment 6:** Add 1-2 group discussion video
- **Author Response 6:** We have uploaded 2 audio recordings (1 individual interview and 1 focus group interview) and their transcripts both in Chinese and English.
- **Comment 7:** Attach all measuring tools files entitled annexure section
- **Author Response 7:** Thank you for this suggestion. All measuring tools both in Chinese and English were attached entitled annexure section.

**Additional Clarifications**

In addition to the above comments, we have attached the interview outline in the annexure section and refined expression in the Conclusion section on page 29 lines 501-512, page 30 lines 522-523 and lines 531-532.
